# Deconstructing the Alternative Lengthening of Telomeres: Integromics Prioritizes Five Master Hubs Dictating Clinical Survival and Therapeutic Vulnerabilities

**DOI:** 10.3390/biology15171531

**Published:** 2026-09-03

**Authors:** Isaac Armendáriz-Castillo, Santiago Guerrero, Andrés Herrera-Yela, Jhommara Bautista, Andrés López-Cortés

**Affiliations:** 1 Latin American Network for Implementation and Validation of Clinical Pharmacogenomics Guidelines (RELIVAF-CYTED), Santiago 8320000, Chile; isaac.arcas@gmail.com; 2 Laboratorio de Ciencia de Datos Biomédicos, Facultad de Ciencias Médicas de la Salud y de la Vida, Universidad Internacional del Ecuador, Quito 170102, Ecuador; sxguerrero@gmail.com; 3 Emerging and Neglected Diseases Group, Health Sciences Faculty, Universidad Internacional SEK (UISEK), Quito 170102, Ecuador; manuel.herrera@uisek.edu.ec; 4 Experimental and Applied Biomedicine Research Group, Health Sciences Faculty, Universidad Internacional SEK (UISEK), Quito 170102, Ecuador; 5 Grupo EXPRELA, CICA-Centro Interdisciplinar de Química y Biología, Departamento de Biología, Faculta de Ciencias, Universidad de la Coruña, Campus de Elviña, 15001 A Coruña, Spain; 6 Cancer Research Group (CRG), Faculty of Medicine, Universidad de Las Américas, Quito 170102, Ecuador; jhommyb@gmail.com

**Keywords:** Alternative Lengthening of Telomeres, transcriptomics, gene signature, survival analysis, DNA repair

## Abstract

The Alternative Lengthening of Telomeres (ALT) pathway drives replicative immortality in aggressive malignancies, particularly sarcomas and gliomas. Clinical ALT stratification has relied on screening for structural ATRX and DAXX mutations. However, this genotypic approach fails to capture the dynamic macro-reprogramming required to sustain ALT. Here, we established and validated a 28-gene transcriptomic signature that captures the ALT-associated transcriptomic phenotype of the ALT phenotype. Using multivariate Cox proportional hazards models and time-dependent ROC analyses, we demonstrate that this signature is a robust, independent predictor of poor overall survival in Sarcoma (SARC) and Lower Grade Glioma (LGG) cohorts, outperforming the prognostic value of traditional ATRX/DAXX mutational status. Genomic mapping revealed this transcriptional synchrony is structurally facilitated by non-random focal clustering on Chromosome 8. To deconstruct the machinery driving this lethal phenotype, we employed an integromic approach, synthesizing protein–protein and metabolic flux networks. Topological algorithms prioritized five indispensable hubs: TP53, ATM, ATR, PCNA, and UBE2I. Gene–metabolite profiling identified PCNA as a bottleneck funneling extreme deoxyribonucleotide (dNTP) demand to sustain break-induced telomeric recombination. To translate these vulnerabilities into actionable treatments, we mapped these hubs to a precision pharmacological network. We propose a multi-targeted strategy combining FDA-approved PARP inhibitors to exploit ATR-mediated synthetic lethality, alongside antimetabolites to induce nucleotide starvation. This study redefines ALT risk stratification and provides a data-driven framework to target and treat resistant ALT-positive tumors.

## Simple Summary

Certain aggressive cancers, such as sarcomas and brain gliomas, achieve cellular “immortality” by lengthening their telomeres using a survival mechanism called ALT. Currently, researchers look for specific DNA mutations to detect ALT, but this method is often insufficient for accurately predicting patient outcomes. In this study, we developed a new diagnostic signature based on the activity of 28 specific genes. We demonstrated this tool provides improved prognostic discrimination of tumor aggressiveness and patient survival than traditional mutation tests. Furthermore, we mapped how these genes interact, discovering they consume high amounts of cellular energy and DNA building blocks to keep the cancer alive. By understanding this machinery, we identified existing FDA-approved drugs, specifically PARP inhibitors and antimetabolites, that are predicted to inhibit this cancer survival engine through potential metabolic starvation and DNA damage. This research offers a robust prognostic framework to stratify high-risk tumors and provides an in silico hypothesis-generating framework.

## 1. Introduction

Telomere maintenance allows cancer cells to evade senescence and achieve replicative immortality [1]. While most tumors reactivate telomerase, approximately 10–15% rely on a telomerase-independent mechanism known as Alternative Lengthening of Telomeres (ALT) [2,3]. Prevalent in aggressive malignancies of mesenchymal and neuroepithelial origin, such as sarcomas and lower-grade gliomas [4], this phenotype relies on homologous recombination (HR) processes to physically extend chromosome ends [5]. ALT activation drives high genomic instability and correlates with aggressive disease progression, poor prognosis, and resistance to standard therapies [6].

ALT research has predominantly focused on its isolated role as a compensatory DNA repair mechanism, driven primarily by the formation of ALT-associated Promyelocytic Leukemia Nuclear Bodies (APBs) [7]. However, our understanding of how this macromolecular machinery reprograms the biological and systemic building of the entire cell remains limited [8]. Sustaining the ALT phenotype demands a higher metabolic toll and a restructuring of protein interaction networks that have yet to be mapped in an integrative manner [5,9].

To address this critical knowledge gap, our research group has progressively established the molecular and metabolic foundations of the ALT mechanism through large-scale bioinformatic approaches. Initially, through a pan-cancer genomic analysis using The Cancer Genome Atlas (TCGA) cohorts, we evaluated hundreds of telomere maintenance genes, successfully identifying a strong panel of highly mutated genes as fundamental molecular markers for ALT [10]. Subsequently, by evaluating multi-omic alterations, we translated the underlying interactome of APBs, identifying 13 key proteins that exhibit distinctive mutational patterns, physical interactions, and functional enrichment across multiple cancer types [11]. Most recently, we demonstrated that these proteins and genetic signatures do not operate in a biochemical vacuum but interact directly with the cellular microenvironment, prioritizing pathways such as purine metabolism, cysteine/methionine metabolism, and nicotinate/nicotinamide metabolism as highly vulnerable metabolic axes in pan-cancer cohorts [12].

Building upon our previous independent findings, which separately mapped the genomic drivers and the APB physical interactome, we propose that the convergence of the ALT phenotype have predominantly been analyzed as separate entities. In the present study, we propose that the convergence of these multiple layers of biological data through an integromics approach is essential to filter biological noise and reveal the true hierarchy of the ALT network [5]. We postulate that the alternative elongation machinery is centralized around a select number of master hubs that simultaneously govern mechanical DNA repair and the immense metabolic flux required to sustain tumor immortalization [13].

The aim of this research is to integrate the transcriptomic profiles, APB structural interactomes, and metabolic networks previously established in our work to validate the functional cores of the ALT phenotype. Through this integrative model, we prioritize five structural and regulatory hubs (*TP53*, *ATM*, *ATR*, *PCNA*, and *UBE2I*) and map their nucleotide and energetic dependencies. Finally, we translate these molecular discoveries into an actionable clinical strategy by designing a precision pharmacological network, proposing the repurposing of FDA-approved agents to induce synthetic lethality and metabolic starvation in the most lethal ALT-positive tumors.

## 2. Materials and Methods

### 2.1. Transcriptional Co-Expression and Prognostic Survival Analysis

To elucidate the clinical and transcriptional dynamics of the ALT molecular machinery, a curated signature of 28 core genes was systematically evaluated. This signature was derived from our previous foundational studies, integrating the 20 ALT-related genes prioritized through pan-cancer genomic screening [10] and the 13 key proteins identified within the ALT-associated PML nuclear body (APB) interactome [11]. By merging these two specialized panels and accounting for the five overlapping genetic components between them, we consolidated a 28-gene module encompassing DNA damage response (DDR), telomere maintenance, and replicative stress.

Transcriptional co-expression patterns within this unified signature were initially assessed by computing pairwise Pearson correlation coefficients. The resulting correlation matrix was subjected to hierarchical clustering and visualized as a heatmap to identify functional modules and regulatory clusters across pan-cancer cohorts. The complete pan-cancer TCGA RNA-seq matrix encompassing primary tumor samples across all 33 cancer types was used to construct the co-expression heatmap.

The clinical prognostic value of the 28-gene panel was analyzed across 33 human cancer types from The Cancer Genome Atlas (TCGA) project [14,15] utilizing the GEPIA 2.0 (Gene Expression Profiling Interactive Analysis) platform [16]. Within the “Survival Map” module, patients were stratified into high- and low-expression cohorts based strictly on the median gene expression threshold (Cutoff-High = 50%, Cutoff-Low = 50%). The pan-cancer prognostic impact was evaluated for both Overall Survival (OS) and Disease-Free Survival (DFS) and visualized as heatmaps.

To validate the clinical vulnerability observed in the pan-cancer screening, specific gene–disease pairs demonstrating the highest risk profiles were selected for detailed Kaplan–Meier survival analysis. We focused on the four most susceptible cohorts: Mesothelioma (MESO), Sarcoma (SARC), Adrenocortical Carcinoma (ACC), and Lower Grade Glioma (LGG). Patients were stratified into high-expression (red) and low-expression (blue) groups using the median expression cutoff. Hazard ratios (HR) and 95% confidence intervals were calculated using Cox proportional hazards regression, and statistical significance was assessed via the Mantel–Cox log-rank test. For all analyses, statistical significance was defined at *p* < 0.05. The initial pan-cancer survival screening across 33 TCGA datasets was explicitly designated as an exploratory, hypothesis-generating phase. Therefore, nominal *p*-values derived from the log-rank test were utilized strictly to cast a wide net and identify candidate tumor cohorts (such as SARC and LGG) for subsequent, rigorous, independent multivariable validation.

### 2.2. Bioinformatic Signature Development and Clinical Prognostic Modeling

Clinical and molecular data for the sarcoma (TCGA-SARC) and lower-grade glioma (TCGA-LGG) cohorts were retrieved from the TCGA Pan-Cancer Atlas via the cBioPortal (https://www.cbioportal.org/) [17,18,19]. For each cohort, three files were downloaded: (i) clinical data (containing Overall Survival (Months), Overall Survival Status, Diagnosis Age/Age, and Sex/Gender), (ii) mRNA expression z-scores relative to diploid samples (RNA Seq V2 RSEM, transposed matrix), and (iii) somatic mutation data. Sample IDs were harmonized to the first 12 characters for consistent matching across files. These two specific cohorts were prioritized for downstream multivariable validation because sarcomas and lower-grade gliomas represent the canonical, most clinically prevalent ALT-driven malignancies, and possess the most robust ATRX/DAXX mutational annotation within the TCGA framework.

The ALT_Score was calculated as the mean of the normalized z-scores of the 28 signature genes (*ARID1A*, *SMG7*, *ATF6*, *SENP5*, *ATR*, *RFC4*, *RAD1*, *CCT5*, *CDKN1A*, *HUS1*, *MCPH1*, *PRKDC*, *TERF1*, *LRATD2*, *NBN*, *RAD54B*, *UBR5*, *RAD21*, *NSMCE2*, *RECQL4*, *FBH1*, *MRE11*, *ATM*, *UBE2I*, *TERF2*, *TP53*, *DNMT1*, *PCNA*). High z-score values were set to ±5 and the final score was standardized (mean = 0, SD = 1) to facilitate interpretation of hazard ratios per standard deviation unit. To ensure this threshold did not bias the prognostic capacity, a sensitivity analysis using unclipped raw expression z-scores (A|LT_Score_Raw) yielded consistent independent prognostic performance and comparable hazard ratios across cohorts.

ATRX/DAXX mutational status was coded as a binary variable (1 = mutated in ATRX or DAXX, 0 = wild-type in both genes) and used as a molecular proxy for ALT activation, given that loss-of-function alterations in these chromatin remodelers are a well-established driver of the ALT phenotype. Mutation calls were extracted directly from the somatic mutation files and binarized accordingly.

To assess the independent prognostic value of the ALT signature while controlling for confounding variables, univariate and multivariate Cox proportional hazards regression models were constructed using the survival (v3.5-0) and survminer (v0.4.9) packages in R (v4.5.3). The survival endpoint was defined as time to death using Surv (Overall_Survival_Months, Overall_Survival_Status), where Overall Survival Status was recoded as 1 (DECEASED) or 0 (LIVING). The multivariate model included the ALT_Score as the primary predictor and was adjusted for Diagnosis Age/Age, Sex/Gender, and ATRX/DAXX mutational status. Hazard ratios (HR), 95% confidence intervals (95% CI), and *p*-values were reported. Model performance was summarized with the concordance index (C-index) and visualized using forest plots generated with ggforest and a custom ggplot2-based implementation to ensure stability and high-resolution output.

To rigorously evaluate the proportional hazards (PH) assumption of the Cox models, scaled Schoenfeld residuals were computed for each covariate and globally using the cox.zph() function from the survival package in R. The independence of residuals relative to time was visually inspected via smoothed spline plots and statistically verified. A non-significant *p*-value (*p* > 0.05) across individual predictors and the global test confirmed that the proportional hazards assumption was strictly satisfied for both cohorts.

To prevent cohort-specific overfitting and maximize cross-cohort reproducibility, the 28-gene signature was constructed as an unweighted z-score sum rather than assigning coefficients from a single regression model. The added prognostic value of the complete 28-gene score over standard structural markers was evaluated using Harrell’s Concordance Index (C-index).

### 2.3. Independent Cohort Validation, Model Discrimination, Likelihood Ratio Testing, and Clinical Utility

Clinical validation and demographic data extraction for the target cohorts (TCGA-SARC and TCGA-LGG) were performed using the cBioPortal database (https://www.cbioportal.org/) to ensure robust clinical annotation. Time-dependent receiver operating characteristic (ROC) curves were generated using the timeROC R package to evaluate the predictive accuracy of the ALT 28-gene signature. The area under the curve (AUC) with 95% confidence intervals (95% CI) was calculated for 1-, 3-, and 5-year overall survival probabilities using Inverse Probability of Censoring Weighting (IPCW).

To rigorously test whether the ALT signature provides incremental prognostic value over baseline clinical parameters (age and gender), nested Cox proportional hazards models were compared. Harrell’s Concordance Index (C-index) and its corresponding 95% CIs were estimated for both the clinical model alone and the combined model (clinical covariates + ALT signature) using bootstrap resampling (B = 1000). The statistical improvement in model fit was formally evaluated using the Likelihood Ratio Test (LRT), reporting Chi-square (X^2^) statistics and *p*-values.

Furthermore, model calibration at the 36-month (3-year) survival mark was evaluated via bootstrap-corrected calibration curves to assess agreement between predicted and observed survival. Finally, Decision Curve Analysis (DCA) was conducted using the dcadcx package in R to quantify the net clinical benefit of incorporating the ALT transcriptomic signature across a range of threshold probabilities.

### 2.4. Chromosomal Localization and Genomic Mapping

Precise genomic coordinates (GRCh38/hg38 assembly) for the 28 prioritized ALT-associated genes were retrieved from the NCBI [20,21] and Ensembl [22] databases. The spatial organization of these genes across the human karyotype was visualized by constructing a genomic ring (Circos) map using the circlize package in the R statistical environment. Chromosomes were arranged sequentially along the outer perimeter, incorporating standard Giemsa-stained (G-band) cytological banding for structural context. The specific loci of the target genes were plotted as points in the inner track (axis scales omitted for visual clarity) to determine whether the ALT transcriptomic module is driven by localized structural variations or distributed pan-genomic regulatory networks.

To statistically evaluate the non-random genomic distribution of the co-expressed genes visualized in the Circos plot, a hypergeometric enrichment test was performed comparing the observed chromosomal localization against the expected genomic background distribution.

### 2.5. Functional Annotation and Pathway Enrichment Analysis

To characterize the biological functions and molecular signaling pathways governed by the 28-gene ALT signature, functional enrichment analysis was performed in the R statistical software (v4.5.3) using the clusterProfiler package. Gene Ontology (GO) analysis was executed focusing on the Biological Process (BP) ontology. Simultaneously, Kyoto Encyclopedia of Genes and Genomes (KEGG) pathway analysis was performed. Statistical significance was evaluated using the Benjamini–Hochberg false discovery rate (FDR) correction, and only terms/pathways with an adjusted *p*-value (*p*.adjust) < 0.05 were retained. Results were visualized as dot plots using the ggplot2 package.

### 2.6. Protein–Protein Interaction (PPI) Network Construction and Topological Analysis

A Protein–Protein Interaction (PPI) network of the 28-gene ALT signature was constructed using interaction data retrieved from the STRING database (v12.0) [23]. Only high-confidence interactions (combined score > 0.700) from experimental, database, textmining, and co-expression sources were included. The resulting interaction matrix was imported into Cytoscape (v3.10.4) for visualization and topological analysis. Degree centrality was calculated using the built-in NetworkAnalyzer tool to identify network hubs. Node size was mapped proportionally to degree centrality, while node fill color was mapped to a continuous gradient of Log_10_(*p*-value) derived from survival analysis, enabling simultaneous evaluation of structural importance and clinical relevance.

### 2.7. Integromic Gene–Metabolite Network Construction

To analyze the metabolic rewiring dictated by the ALT machinery, a gene–metabolite interaction network was constructed using a dual-platform approach. The 28-gene signature was first analyzed in the Network Explorer module of MetaboAnalyst 6.0 [24] to map associated endogenous metabolites and biochemical pathways. These interactions were cross-referenced and expanded using the STITCH database (v5.0), retaining only high-confidence interactions (confidence score > 0.700) from experimental, database, and text-mining sources. The integrated bipartite network (proteins vs. metabolites) was imported into Cytoscape (v3.10.4). Nodes were distinguished by shape and color (proteins vs. chemical compounds), and degree centrality was computed with NetworkAnalyzer to identify the most heavily trafficked metabolic bottlenecks.

### 2.8. Pharmacological Network Analysis and Drug Repurposing

To translate molecular and metabolic vulnerabilities into actionable therapeutic strategies, a drug–gene interaction network was constructed by querying the master hubs of the ALT signature against the Drug–Gene Interaction Database (DGIdb, v5.0) [25]. The search was restricted to FDA-approved antineoplastic and immunomodulating agents. Drug classes prioritized for synthetic lethality and metabolic starvation included DNA-damaging/alkylating agents, PARP inhibitors, topoisomerase inhibitors, and antimetabolites. The curated drug–target interaction matrix was imported into Cytoscape (v3.10.4). Edge width was continuously mapped to the documented interaction score to visually represent the strength of each pharmacological association.

## 3. Results

### 3.1. The ALT Signature Operates as a Coordinated Transcriptional Module Driving Poor Prognosis

**Figure 1:**
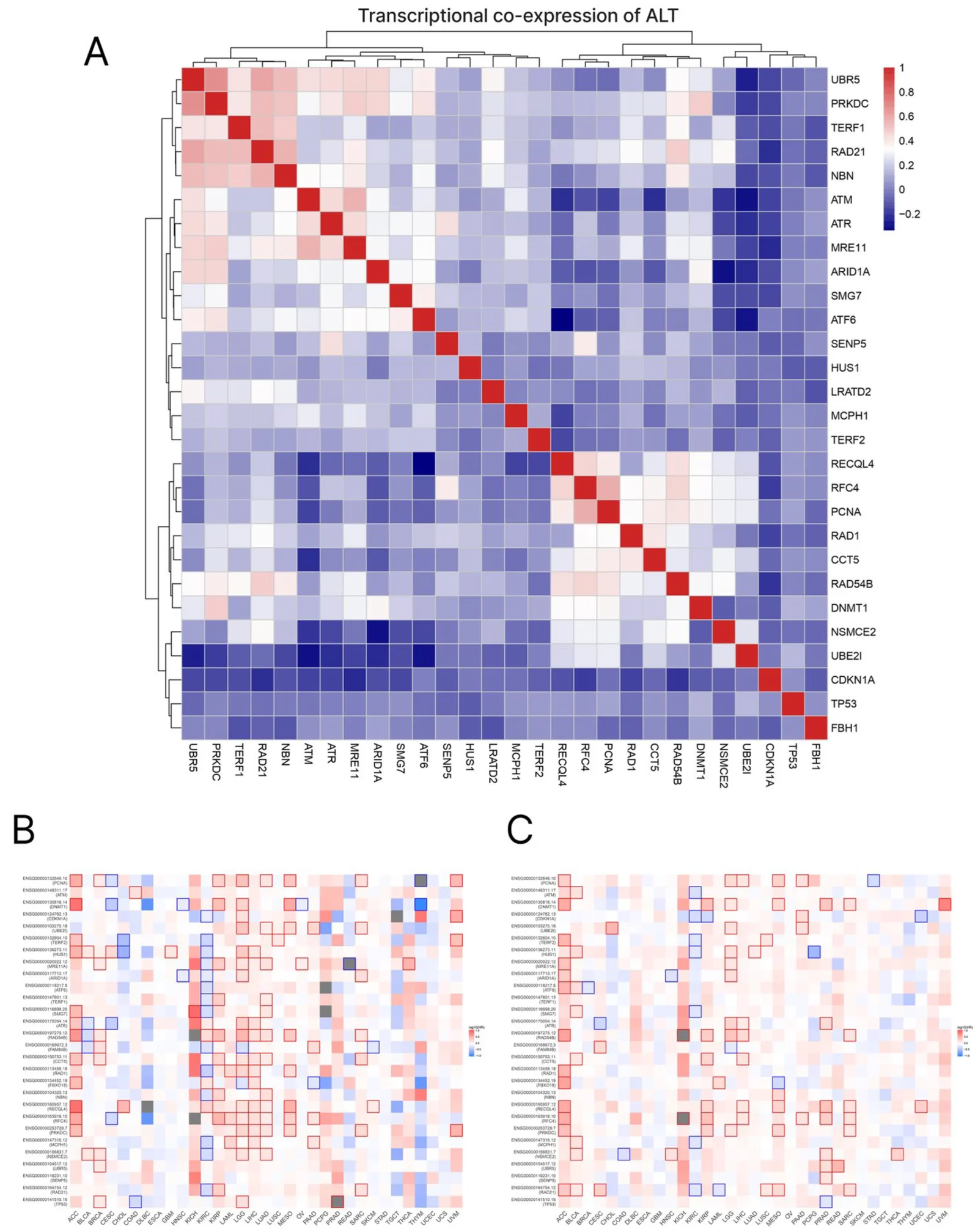
Transcriptional co-expression and pan-cancer prognostic landscape of the 28-gene ALT signature. (**A**) Hierarchical clustering heatmap displaying the pairwise transcriptional co-expression of the 28 core genes associated with the Alternative Lengthening of Telomeres (ALT) mechanism. The color gradient represents the Pearson correlation coefficient, with dark red indicating a strong positive correlation and dark blue indicating a negative correlation. The complete pan-cancer TCGA RNA-seq matrix encompassing primary tumor samples across all 33 cancer types was used to construct the co-expression heatmap. (**B**) Pan-cancer Overall Survival (OS) map across 33 TCGA tumor types. (**C**) Pan-cancer Disease-Free Survival (DFS) map. In the survival maps (**B**,**C**), the color intensity reflects the Hazard Ratio (HR) on a logarithmic scale (red = high risk, blue = low risk). Squares highlighted with a solid border denote a statistically significant impact on patient survival (Log-rank test, *p* < 0.05).

To determine whether the 28 identified markers operate independently or as a synergistic network, we evaluated their transcriptional co-expression (Figure 1A). Hierarchical clustering revealed a molecular structure characterized by strong positive correlations (red modules) among primary DDR and telomere maintenance hubs (e.g., ATM, ATR, PRKDC, RAD21).

This result suggests a coordinated transcriptional activation required to sustain the high replicative demands of the ALT pathway. Equally, master tumor suppressors, notably TP53 and CDKN1A (p21), exhibited distinct negative correlation patterns with replicative factors, highlighting the intrinsic regulatory feedback loops where the suppression of cell cycle checkpoints is permissive for continuous telomeric recombination.

To translate these transcriptional patterns into clinical relevance within an exploratory pan-cancer framework, we assessed their preliminary prognostic impact across 33 TCGA tumor types. The pan-cancer survival maps for Overall Survival (OS) (Figure 1B) and Disease-Free Survival (DFS) (Figure 1C) demonstrated that the overexpression of the signature genes predominantly acts as a high-risk factor (HR > 1, red blocks). The deepest and statistically significant prognostic deterioration was observed in Lower Grade Glioma (LGG), Sarcoma (SARC), Adrenocortical Carcinoma (ACC), and Mesothelioma (MESO).

### 3.2. Kaplan–Meier Validation Highlights Specific Vulnerabilities in ALT-Prevalent Tumors

**Figure 2:**
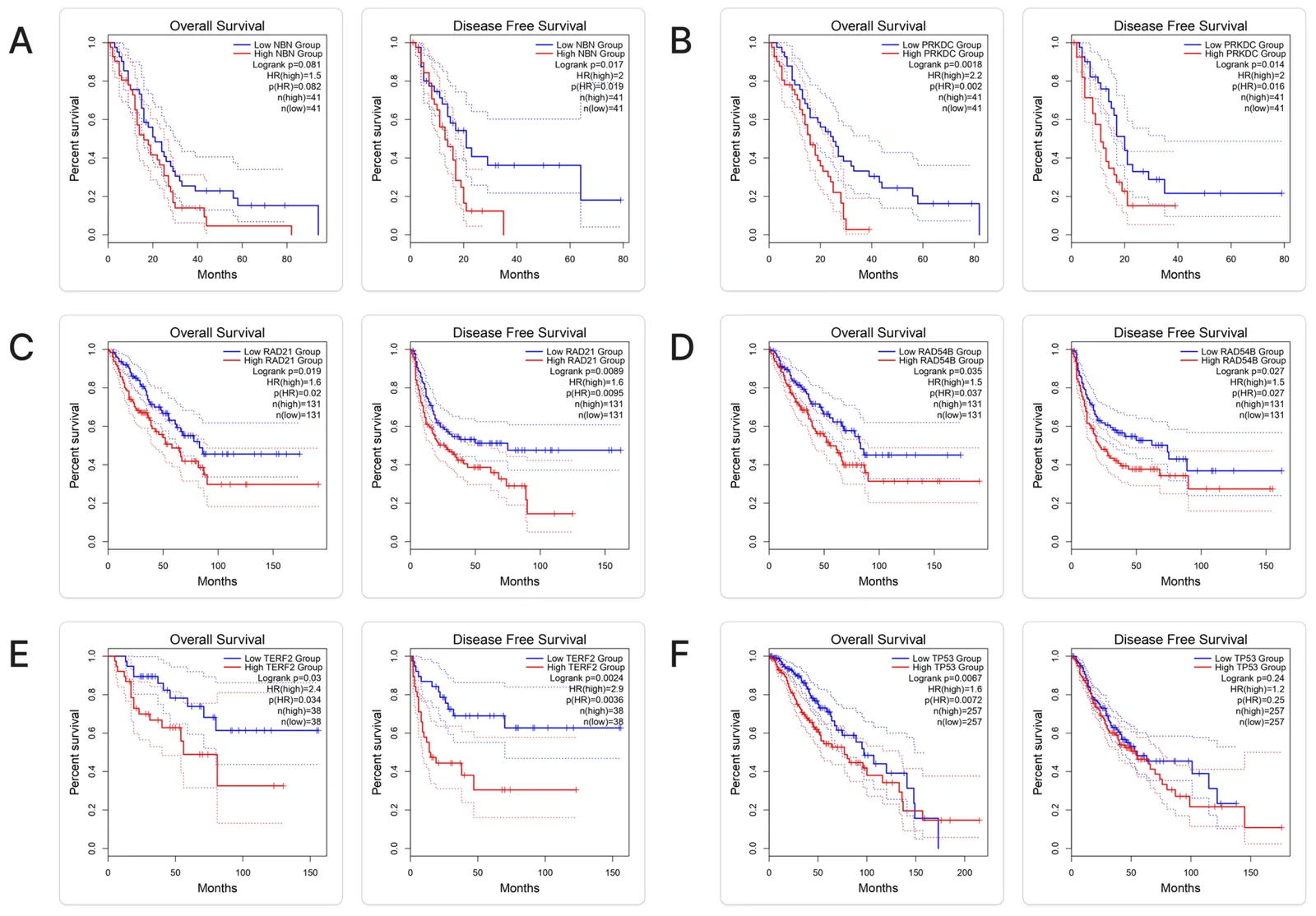
Kaplan–Meier survival validation of prioritized ALT hub genes in highly susceptible clinical cohorts. Survival curves stratifying patients into high-expression (red line) and low-expression (blue line) groups based on median gene expression cutoffs. (**A**,**B**) Overall Survival (OS) and Disease-Free Survival (DFS) for NBN and PRKDC in Mesothelioma (MESO). (**C**,**D**) OS and DFS for RAD21 and RAD54B in Sarcoma (SARC). (**E**) OS and DFS for TERF2 in Adrenocortical Carcinoma (ACC). (**F**) OS and DFS for TP53 in Lower Grade Glioma (LGG). Dotted lines represent 95% confidence intervals. Statistical significance was determined using the Mantel–Cox log-rank test, with hazard ratios (HR) indicating the relative risk of the high-expression cohort.

To validate the pan-cancer screening at the single-gene level, we performed detailed Kaplan–Meier survival analyses on specific, highly deregulated gene–disease pairs within the four most severely impacted cohorts: Mesothelioma (MESO), Sarcoma (SARC), Adrenocortical Carcinoma (ACC), and Lower Grade Glioma (LGG) (Figure 2).

In Mesothelioma (MESO), the overexpression of DNA double-strand break repair components critically reduced patient survival. High expression of PRKDC significantly worsened both OS (*p* = 0.0018, HR = 2.2) and DFS (*p* = 0.014, HR = 2) (Figure 2B), while NBN overexpression significantly accelerated disease relapse (DFS *p* = 0.017) (Figure 2A).

Similarly, in Sarcoma (SARC) the upregulation of recombination and cohesion factors drastically stratified patients into poor prognostic groups. High levels of RAD21 (Figure 2C) and RAD54B (Figure 2D) were both significantly associated with a marked decrease in OS and DFS (*p* < 0.05), confirming that elevated homologous recombination activity drives tumor aggressiveness.

Furthermore, the overexpression of the shelterin complex component TERF2 in ACC (Figure 2E) resulted in a severe reduction in both OS (*p* = 0.03, HR = 2.4) and DFS (*p* = 0.0024, HR = 2.9). Finally, in Lower Grade Glioma (LGG), elevated expression of TP53 was strongly predictive of diminished Overall Survival (*p* = 0.0067, HR = 1.6) (Figure 2F), showing its complex, often mutated role in central nervous system malignancies. Together, these validations confirm that the hyperactivation of specific ALT-related DDR and telomeric factors directly dictates clinical failure in highly susceptible tumor microenvironments.

### 3.3. Internal Prognostic Validation of the ALT Transcriptomic Signature: A Multivariate Cox Regression Analysis

To evaluate the internal clinical prognostic utility of the 28-gene ALT signature, multivariate Cox proportional hazards models were constructed for both the Sarcoma (SARC) and Lower Grade Glioma (LGG) cohorts. The models were adjusted for patient age, gender, and *ATRX*/*DAXX* mutational status—the classical genomic surrogates for ALT activation. The complete clinical and molecular matrices utilized for these models are provided in Supplementary Tables S1–S6.

#### 3.3.1. Transcriptomic Prognostic Stratification in Sarcoma (SARC)

**Figure 3:**
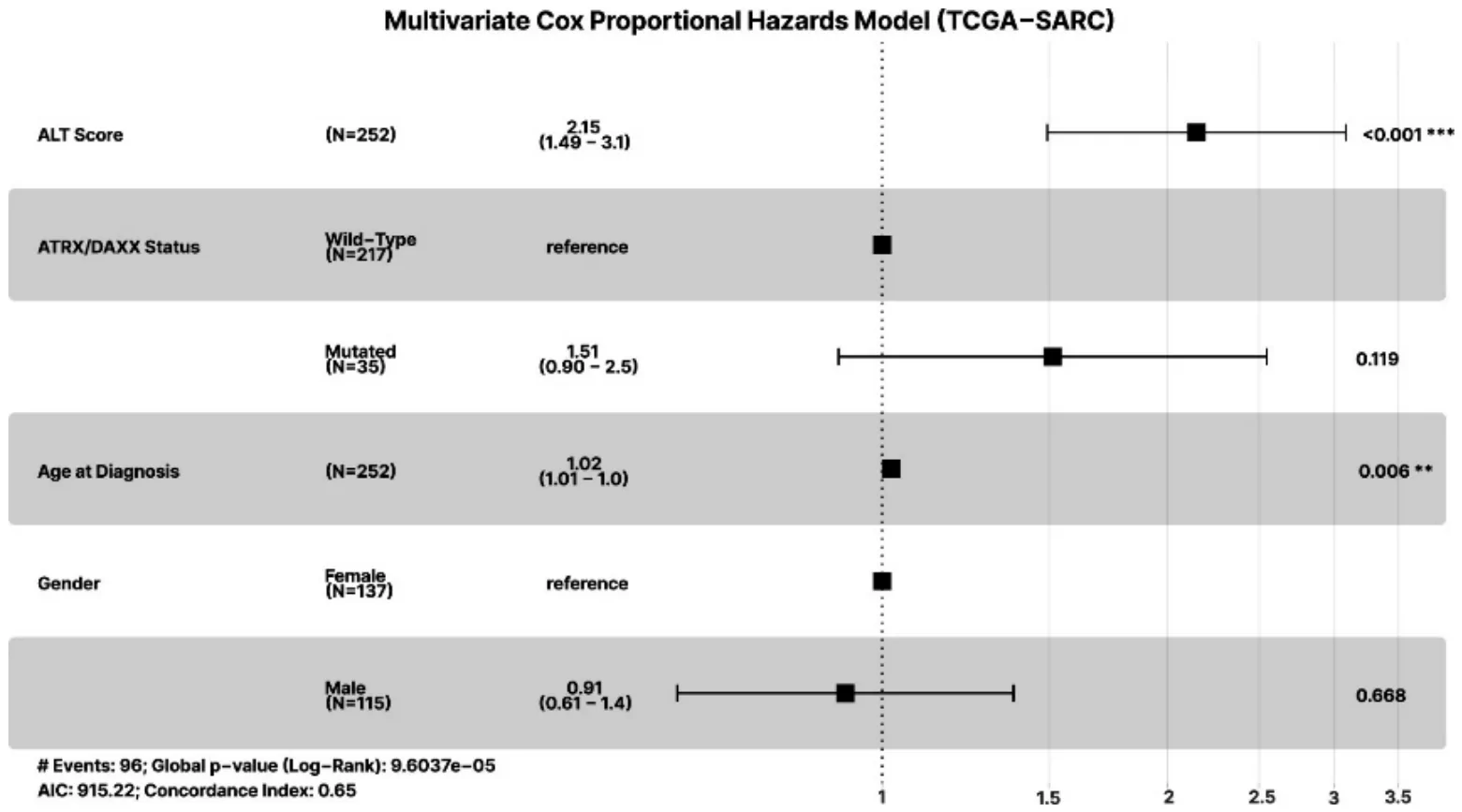
Multivariate Cox proportional hazards analysis of the ALT signature in the TCGA-SARC cohort. The forest plot displays the Hazard Ratios (HR), 95% Confidence Intervals (CI), and *p*-values for the 28-gene ALT Score, *ATRX*/*DAXX* mutational status, age at diagnosis, and gender. The ALT signature demonstrates highly significant independent prognostic value (*p* < 0.001), outperforming traditional mutational markers. The square size represents the precision of the estimate (HR), and horizontal bars indicate the 95% CI. (*** highly significant, ** medium significant).

In the SARC cohort (N = 252, 96 events), the multivariate regression model (Global log-rank *p* = 9.6 × 10^−5^) revealed that the ALT signature score is a strong and independent predictor of poor overall survival (OS). As illustrated in the Forest Plot (Figure 3), elevated expression of the ALT-associated gene module significantly increases the hazard of mortality by over two-fold (HR: 2.15, 95% CI: 1.49–3.1, *p* < 0.001) (Supplementary Table S7).

The ALT signature maintained high statistical significance after adjusting for all covariates. In contrast, the traditional *ATRX*/*DAXX* mutational status failed to reach statistical significance as an independent prognostic factor in this model (HR: 1.51, 95% CI: 0.90–2.5, *p* = 0.119). Age at diagnosis was also identified as a significant, albeit modest, prognostic variable (HR: 1.02, *p* = 0.006), while patient gender exhibited no impact on survival outcomes (HR: 0.91, *p* = 0.668). These findings strongly suggest that the transcriptomic rewiring captured by the ALT signature provides superior prognostic stratification compared to relying solely on the structural loss-of-function of chromatin remodelers.

To test whether the 28-gene score provides superior discrimination compared to traditional markers, we calculated Harrell’s C-index in the TCGA-SARC cohort. The base model using *ATRX/DAXX* mutational status alone yielded a C-index of 0.521, whereas the 28-gene ALT_Score achieved a significantly higher C-index of 0.625 (Supplementary Table S8), confirming its substantial added prognostic value.

Additionally, the proportional hazards assumption for the multivariate SARC model was formally tested using scaled Schoenfeld residuals. The global test demonstrated no departure from proportional hazards (*p* = 0.7735), and all individual covariates fulfilled the assumption ALT_Score: *p* = 0.4012; Diagnosis Age *p* = 0.5167; Sex *p* = 0.5366; ATRX/DAXX status *p* = 0.7461; Tumor Type *p* = 0.4487), confirming the temporal constancy of the estimated hazard ratios across the follow-up period (Supplementary Figure S1).

#### 3.3.2. Validation in Lower Grade Glioma (LGG)

**Figure 4:**
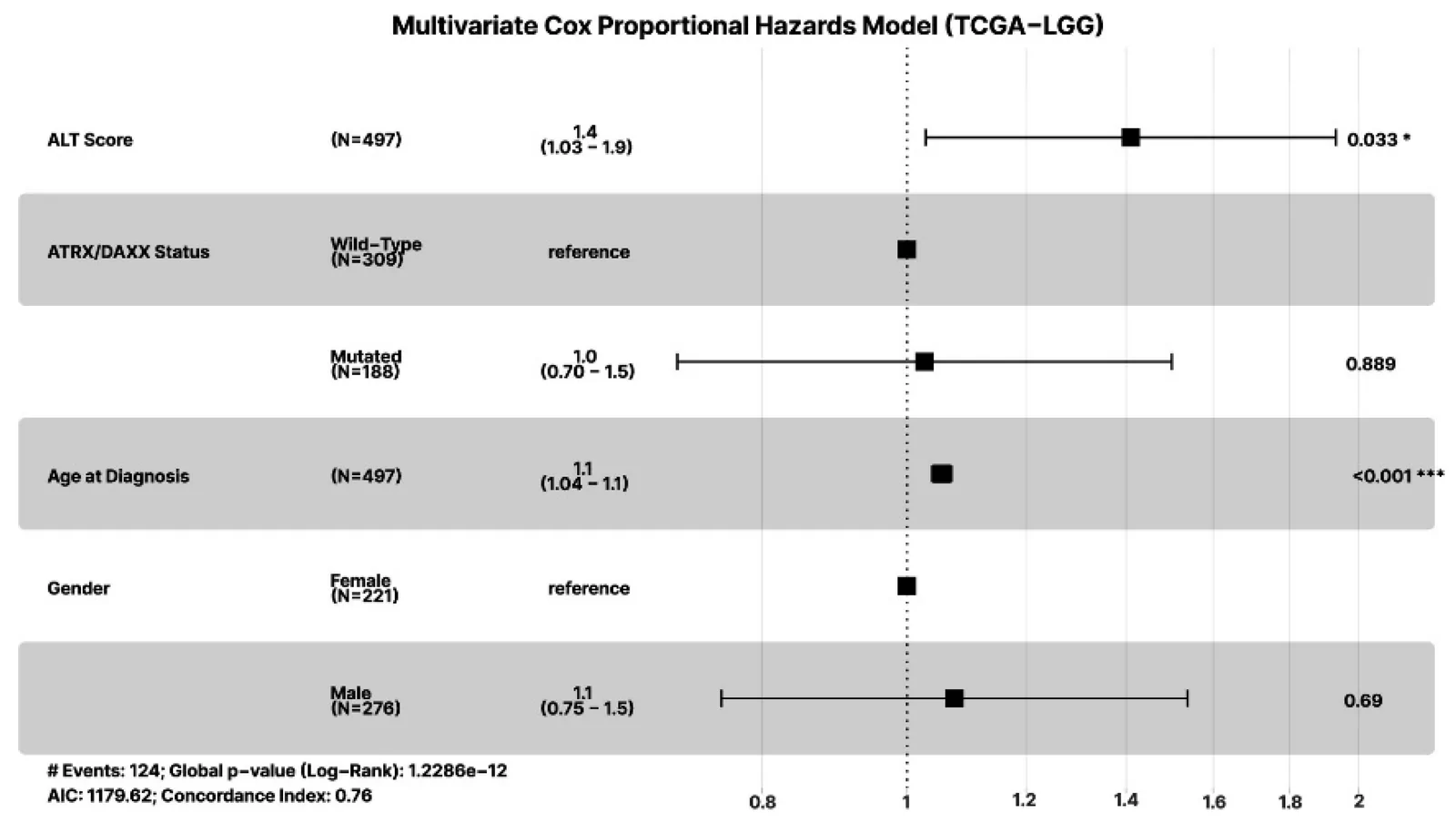
Multivariate Cox proportional hazards analysis of the ALT signature in the TCGA-LGG cohort. The forest plot illustrates the independent prognostic power of the 28-gene ALT Score adjusted for clinical covariates and *ATRX*/*DAXX* mutations. The ALT transcriptomic signature remains a significant predictor of overall survival (*p* = 0.033), confirming its translational value across different highly susceptible ALT-driven cancer types. (*** highly significant, * significant).

The prognostic value of the ALT signature was further validated in the LGG cohort (N = 497, 124 events). The multivariate model (Global log-rank *p* = 1.22 × 10^−12^) confirmed the signature’s predictive value, demonstrating a significant association with increased mortality risk (HR: 1.40, 95% CI: 1.03–1.9, *p* = 0.033) (Figure 4) (Supplementary Tables S9 and S10).

Consistent with the SARC findings, the *ATRX*/*DAXX* mutational status did not show independent prognostic significance (HR: 1.0, 95% CI: 0.70–1.5, *p* = 0.889). This reinforces the hypothesis that the functional activation of the ALT pathway, rather than the mere presence of upstream mutations, is the primary driver of aggressive tumor behavior and poor patient outcomes. As anticipated in neuro-oncology, age at diagnosis emerged as a powerful confounding risk factor (HR: 1.10, *p* < 0.001), which our model successfully adjusted for, ensuring that the predictive value of the ALT score is genuinely independent of demographic variables.

Furthermore, evaluation of scaled Schoenfeld residuals confirmed that the LGG multivariate model strictly complied with the proportional hazard’s assumption. Both the global model (*p* = 0.5819) and all individual predictors (ALT_Score: *p* = 0.9195; Sex: *p* = 0.8935; Diagnosis Age: *p* > 0.05; ATRX/DAXX Status: *p* > 0.05) showed no statistically significant time-dependent variation (*p* > 0.05), validating the structural robustness of the Cox proportional hazards modeling in this cohort (Supplementary Figure S2). Finally, a sensitivity analysis evaluating the multivariate models without z-score clipping confirmed that the prognostic value of the ALT signature remains robust and statistically consistent without this transformation (Supplementary Table S11).

### 3.4. Time-Dependent Predictive Accuracy, Model Discrimination, and Clinical Utility of the ALT Signature

**Figure 5:**
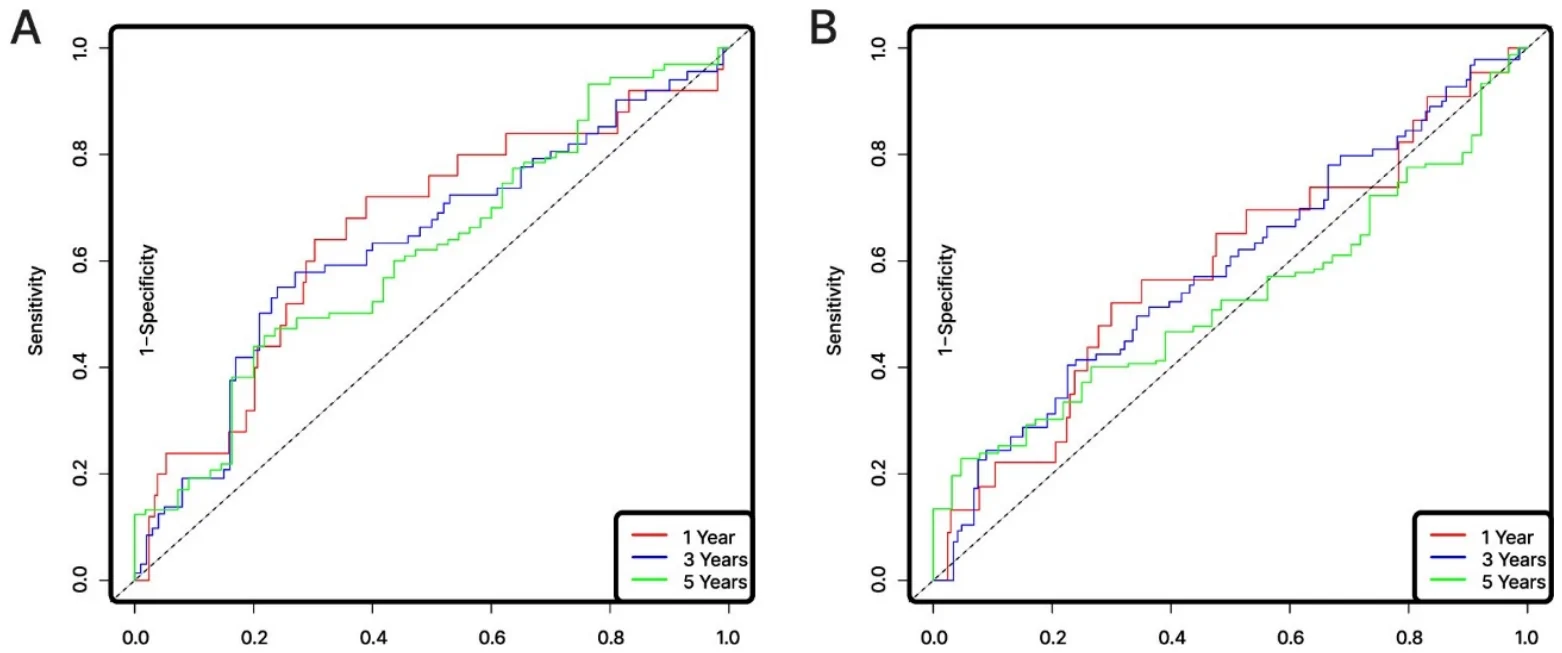
(**A**) Time-dependent Receiver Operating Characteristic (ROC) curve of the ALT signature in the SARC cohort. The graph illustrates the predictive accuracy of the 28-gene ALT score for overall survival at 1 year (red line), 3 years (blue line), and 5 years (green line). The X-axis represents 1—Specificity (False Positive Rate), and the Y-axis represents Sensitivity (True Positive Rate). The signature maintains a stable Area Under the Curve (AUC) ranging from 61.64% to 65.75% across the 5-year longitudinal follow-up. (**B**) Time-dependent Receiver Operating Characteristic (ROC) curve of the ALT signature in the LGG cohort. The predictive performance of the ALT signature for 1-, 3-, and 5-year survival is shown by the red, blue, and green lines, respectively. The analysis demonstrates a baseline discriminative capacity in a highly heterogeneous glioma cohort, complementing its role as an independent prognostic risk factor.

To evaluate the discriminatory capacity and longitudinal predictive accuracy of the 28-gene ALT signature, time-dependent Receiver Operating Characteristic (ROC) curve analysis was conducted. In the TCGA-SARC cohort (N = 252), the ALT signature demonstrated sustained predictive accuracy across all evaluated time points, yielding AUC values of 0.652 (95% CI: 0.533–0.770) at 1 year, 0.632 (95% CI: 0.542–0.722) at 3 years, and 0.619 (95% CI: 0.523–0.715) at 5 years (Figure 5A, Table 1). In the TCGA-LGG cohort (N = 497), time-dependent AUCs were 0.587 (95% CI: 0.459–0.714) at 1 year, 0.572 (95% CI: 0.490–0.654) at 3 years, and 0.520 (95% CI: 0.422–0.617) at 5 years (Figure 5B, Table 1) (Supplementary Figure S3).

To demonstrate that the ALT signature provides significant incremental prognostic performance beyond baseline discriminative capacity (age and gender), we performed comparative model discrimination and Likelihood Ratio Testing (LRT) (Table 1). In TCGA-SARC, adding the ALT score to the clinical baseline increased the C-index from 0.617 (95% CI: 0.551–0.682) to 0.671 (95% CI: 0.610–0.732). The Likelihood Ratio Test confirmed a highly significant improvement in model fit X^2^ = 17.162, *p* = 3.43 × 10^−5^). Similarly, in TCGA-LGG, the combined model achieved a C-index of 0.622 (95% CI: 0.560–0.684) compared to 0.579 (95% CI: 0.511–0.646) for the clinical model alone, with LRT demonstrating statistically significant incremental fit (X^2^ = 9.391, *p* = 0.00218).

Bootstrap-corrected calibration curves at 36 months (3 years) demonstrated satisfactory alignment between predicted survival probabilities and observed outcomes in both cohorts, confirming the reliability of the combined model predictions (Supplementary Figure S4A,C). Furthermore, Decision Curve Analysis (DCA) demonstrated that the combined model (clinical + ALT signature) yielded a superior net clinical benefit over both the “treat-all” and “treat-none” strategies across a wide spectrum of threshold probabilities (Supplementary Figure S4B,D). Collectively, these metrics verify that the 28-gene ALT transcriptomic signature is not only an independent risk factor but also adds quantifiable clinical utility and discrimination power to standard prognostic models.

### 3.5. Genomic Distribution Reveals a Pan-Genomic ALT Network with Pronounced Focal Hotspots

**Figure 6:**
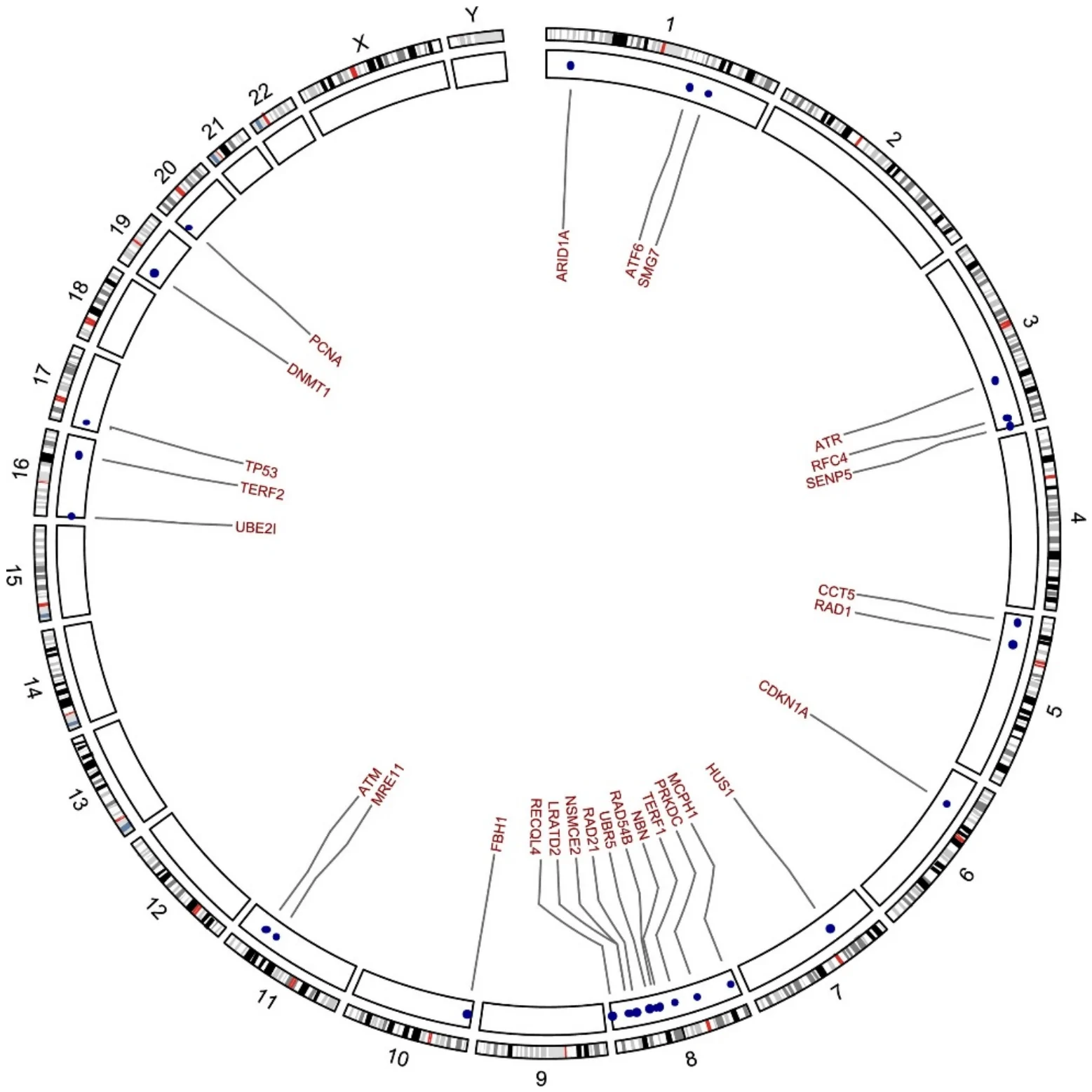
Chromosomal localization and genomic distribution of the 28-gene ALT signature. The Circos plot illustrates the physical mapping of the core ALT-associated genes across the human karyotype (GRCh38/hg38 assembly). The outer ring displays the chromosomes sequentially arranged with standard G-band cytological structural annotations. The inner track marks the specific genomic loci of the 28 target genes. The topological analysis highlights a pan-genomic regulatory network with a pronounced focal clustering on Chromosome 8 (hosting 10 core genes) and a secondary hotspot on Chromosome 3, suggesting a structural basis for the synchronized co-expression of the ALT machinery.

To clarify the structural genomic architecture underlying the coordinated expression of the ALT machinery, the precise chromosomal coordinates of the 28 core genes were mapped across the human karyotype utilizing a Circos plot (Figure 6). The topological mapping demonstrated that the ALT genomic signature is broadly distributed across multiple distinct chromosomes (e.g., chromosomes 1, 3, 10, 16, 17, and 20). This widespread physical distribution indicates that the ALT phenotype is primarily composed of complex, trans-acting transcriptomic networks rather than emerging merely from a single, localized genomic amplification event.

However, the topological analysis revealed a non-random focal clustering of these critical vulnerabilities. Most notably, a dense aggregation of the ALT molecular machinery is localized on Chromosome 8. Ten of the 28 core genes—specifically *MCPH1*, *PRKDC*, *TERF1*, *NBN*, *RAD54B*, *UBR5*, *RAD21*, *NSMCE2*, *RECQL4*, and *LRATD2*—are tightly mapped to this specific chromosome, representing over a third of the entire transcriptomic signature. Furthermore, a secondary structural hotspot was identified on Chromosome 3, which harbors an additional cluster of three essential ALT-associated genes, while core DNA double-strand break (DSB) repair components, such as *ATM* and *MRE11*, remain co-localized on Chromosome 11, and *FBH1* is distinctly mapped to Chromosome 10.

Visual inspection of the Circos plot revealed a striking clustering of the signature genes on Chromosome 8. To confirm that this genomic organization was non-random, a formal hypergeometric test was conducted. Out of the 28 genes in the signature, 10 mapped to Chromosome 8, representing a highly significant regional enrichment (*p* < 1.91828 × 10^−8^). This indicates a statistically robust coordinated spatial expression.

### 3.6. In Silico Functional Validation: The ALT Signature Is Driven by Double-Strand Break Repair and Telomeric Recombination

**Figure 7:**
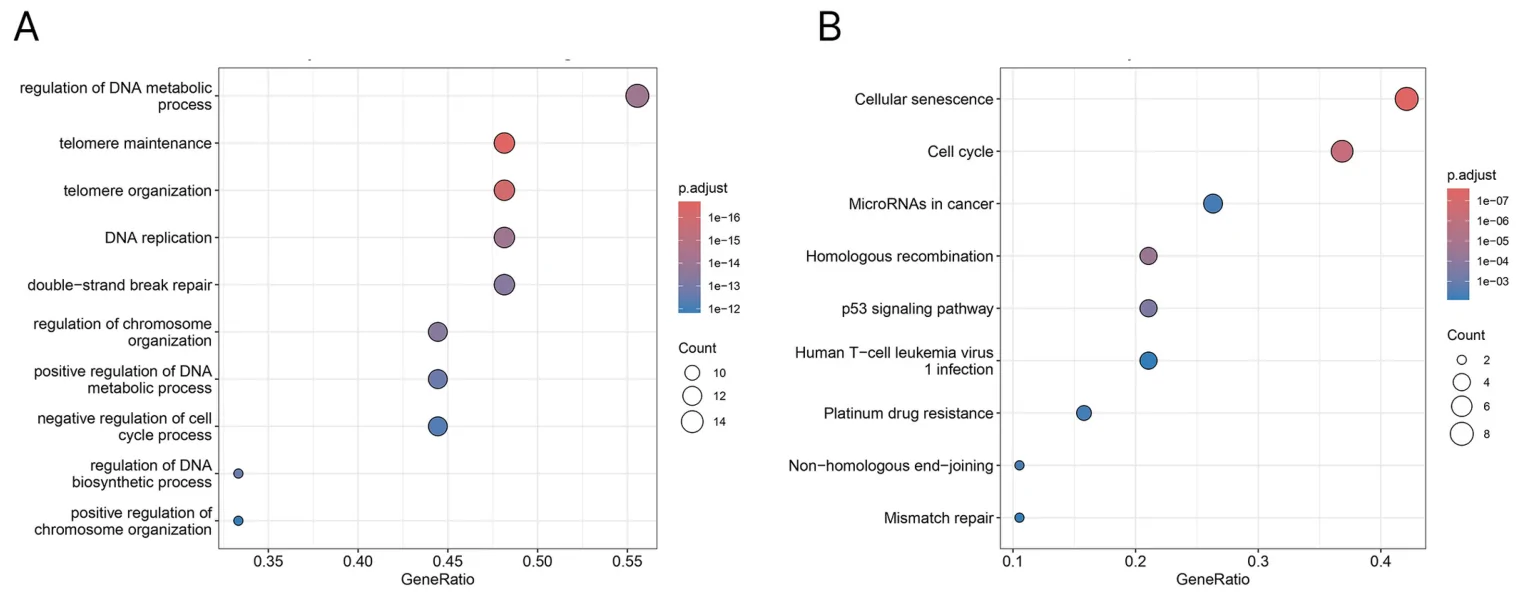
Functional enrichment analysis of the 28-gene ALT signature. (**A**) Gene Ontology (GO) Biological Process analysis highlighting the significant enrichment of terms related to telomere maintenance, DNA replication, and double-strand break repair. (**B**) Kyoto Encyclopedia of Genes and Genomes (KEGG) pathway analysis revealing the hijacking of cellular senescence, cell cycle, and homologous recombination networks. In both panels, the Y-axis represents the enriched terms, and the X-axis indicates the GeneRatio. Node size corresponds to the number of genes involved, and the color gradient (blue to red) reflects statistical significance (*p*.adjust).

To determine the collective biological function of our prioritized gene signature and provide a mechanistic rationale for its clinical prognostic value, we performed an in silico Gene Ontology (GO) and KEGG enrichment analysis (Figure 7).

The GO Biological Process analysis (Figure 7A) definitively confirmed the core mechanical nature of the signature. The most highly significant enriched terms (red nodes, *p* adjust < 1 × 10^−14^) were heavily concentrated on telomere dynamics (“telomere maintenance”, “telomere organization”) and genomic stability (“regulation of DNA metabolic process”, “DNA replication”). “Double-strand break repair” emerged as a dominant term. Since the Alternative Lengthening of Telomeres (ALT) relies strictly on break-induced replication rather than telomerase activity to elongate chromosome ends, this functional enrichment serves as a strong in silico validation, physically verifying our 28-gene signature as the primary molecular engine driving the ALT phenotype.

Furthermore, the KEGG pathway analysis (Figure 7B) provided deep insights into the regulatory networks hijacked by these tumors. The genes were highly enriched in the “Cellular senescence” and “Cell cycle” pathways, highlighting the ability of ALT-positive cells to bypass the Hayflick limit and achieve cellular immortalization. Consistent with the GO findings, “Homologous recombination” and “Non-homologous end-joining” were significantly enriched, reaffirming the absolute dependency of ALT tumors on DNA repair machineries. Notably, the KEGG analysis also revealed a highly significant enrichment in the ‘p53 signaling pathway’ and ‘Platinum drug resistance’ networks, exposing these signaling axes as critical components of the ALT survival strategy and setting the stage for targeted therapeutic interventions.

### 3.7. Topological Network Analysis Reveals a Highly Connected Hub-and-Spoke Architecture Linked to Clinical Survival

**Figure 8:**
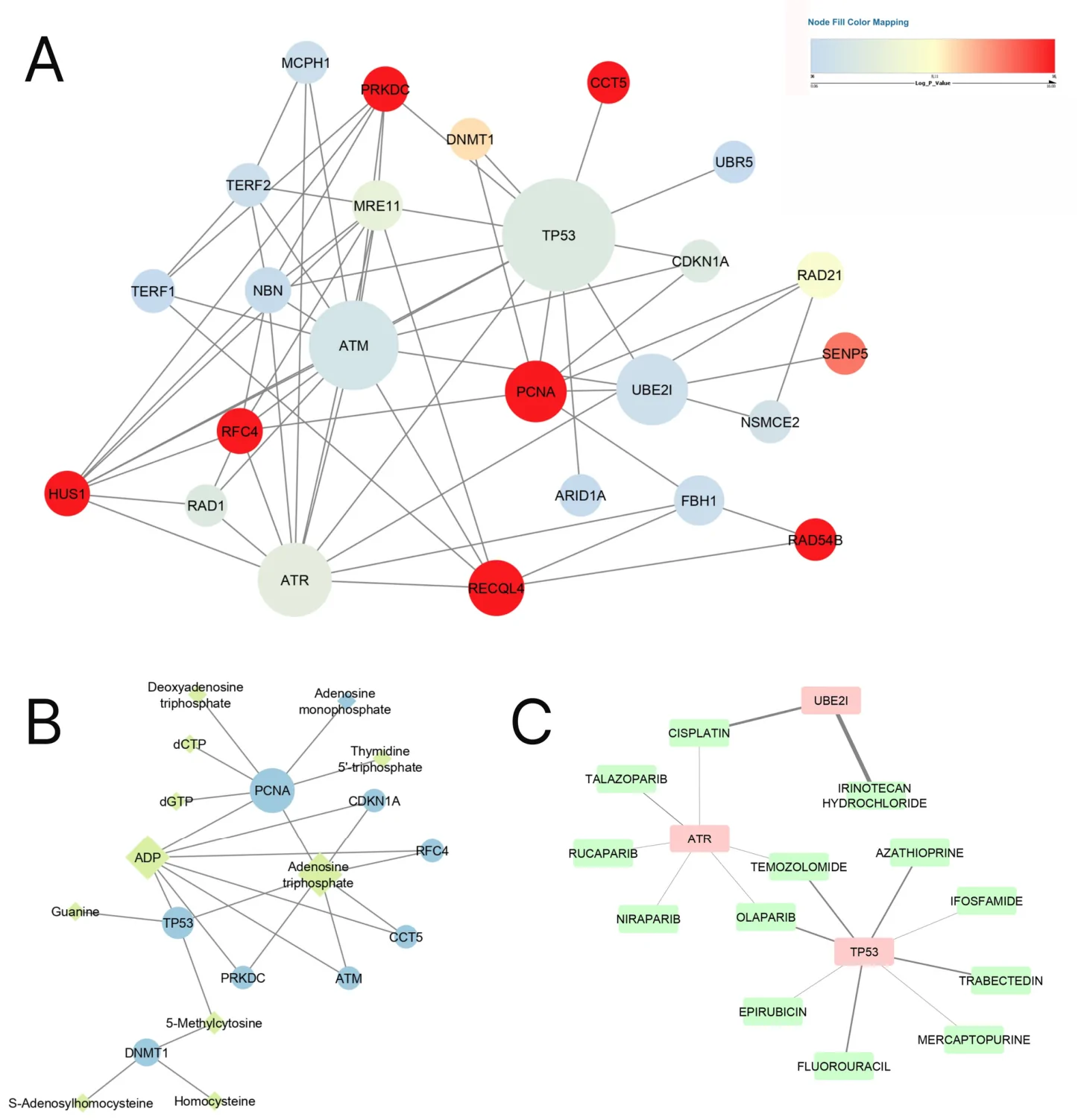
(**A**) Protein–Protein Interaction (PPI) network and topological survival mapping. The interactome reveals a highly cohesive macromolecular structure governed by central hubs (TP53, ATM, ATR). Node size is proportional to degree centrality (number of connections). Node fill color is continuously mapped to the Log_10_(*p*-value) derived from the multivariate Cox survival analysis, with intense red indicating the highest clinical significance (e.g., PRKDC, HUS1, PCNA, RECQL4). This visualizes the intersection of structural network importance and clinical prognostic risk. (**B**) Integromic gene–metabolite network of the ALT machinery. A bipartite interaction network demonstrating the biochemical dependencies of the ALT signature. Blue circular nodes represent the core ALT proteins, while light-green diamond nodes represent chemically interacting metabolites. The network highlights two high biochemical sinks: an energetic axis centered around ATP/ADP interacting with DNA damage kinases, and a structural nucleotide axis (dATP, dCTP, dGTP, dTTP) exclusively funneled through PCNA. (**C**) Targeted pharmacological network for drug repurposing in ALT+ tumors. The drug–gene interactome centers on three master vulnerabilities of the ALT signature: TP53, ATR, and UBE2I (red nodes). Green nodes represent FDA-approved antineoplastic agents. Edge thickness corresponds to the interaction confidence score from the DGIdb database. The network maps out targeted strategies including PARP inhibition (synthetic lethality via ATR), antimetabolite starvation (via TP53), and alkylating/topoisomerase damage (via UBE2I and bridging nodes).

While transcriptional co-expression indicates coordinated regulation, the physical execution of the ALT phenotype requires the direct assembly of protein complexes. To visualize this structural framework and validate its clinical relevance, we constructed a Protein–Protein Interaction (PPI) network of the 28 core targets (Figure 8A). The resulting interactome displayed a highly cohesive and interconnected topology, confirming that these markers form a functional macromolecular machine rather than operating in isolated pathways.

Topological analysis based on degree centrality (indicated by node size) identified TP53, ATM, and ATR as the absolute structural hubs of the network. The high connectivity of these three master kinases physically bridges multiple sub-modules, positioning them as the central signaling coordinators of the ALT stress response.

To understand this phenotype mapping the statistical significance derived from our prognostic survival analysis to the node color (continuous red gradient representing Log_10_(*p*-value), the network revealed a secondary layer of critical effectors. Proteins heavily involved in direct DNA replication and double-strand break repair—specifically PRKDC, HUS1, RFC4, CCT5, and RECQL4—displayed intense red coloration, indicating peak clinical significance. Notably, PCNA emerged as a central predicted structural bridge between the TP53 regulatory hub and the UBE2I/RAD21 sub-network. This topology demonstrates that the most clinically detrimental genes in our Cox models are also the central structural pillars bridging the DNA damage sensors (ATM/ATR) to the executioner repair complexes.

### 3.8. Integromic Profiling Exposes a High Energetic and Nucleotide Metabolic Demand in the ALT Phenotype

To understand the biochemical cost of sustaining continuous, telomerase-independent telomere elongation, we performed an in silico integration of our genomic data with metabolomic interaction networks. The resulting gene–metabolite interactome (Figure 8B) illustrates the deep metabolic rewiring composed of the core protein machinery.

Topological analysis of this bipartite network identified two distinct, highly centralized metabolic hubs. First, the energy carrier axis (ATP and ADP) forms the densest core of the network, directly interacting with master regulators and structural kinases including TP53, ATM, PRKDC, CCT5, and RFC4. This pervasive connectivity strongly suggests that the chronic DNA damage response and the assembly of repair complexes at telomeric ends in ALT+ tumors are energetically hypersensitive processes, creating a high localized ATP sink.

Second, we observed a structural convergence on DNA synthesis building blocks. The proliferating cell nuclear antigen (PCNA) emerged as the structural bridge between the proteome and the nucleotide flux. PCNA is heavily and directly connected to the entire pool of deoxyribonucleotides (dATP, dCTP, dGTP, and dTTP). Since the ALT mechanism relies on break-induced homologous recombination to physically synthesize high tracts of new telomeric DNA, this interaction node validates PCNA as a potential primary funnel for the tumor’s extreme nucleotide demand. Additionally, the network revealed a specialized epigenetic sub-module coordinated by DNMT1, tightly coupled with methionine cycle metabolites (Homocysteine, S-Adenosylhomocysteine) and 5-Methylcytosine. Ultimately, this integromic network exposes the biochemical vulnerabilities of ALT+ cells: a targetable dependency on nucleotide availability and energetic flux.

### 3.9. In Silico Pharmacological Repurposing Identifies Synthetic Lethal and Metabolic Vulnerabilities

Having established through our in silico validations that the ALT phenotype relies heavily on continuous DNA double-strand break repair (Figure 7) and demands a high influx of nucleotide building blocks (Figure 8B), we sought to identify therapeutic agents capable of exploiting these specific bottlenecks. We constructed a targeted drug–gene interaction network centered on the master regulatory hubs: ATR, TP53, and UBE2I (Figure 8C).

The resulting interactome mapped out three distinct pharmacological strategies to sabotage the ALT machinery:

- Induction of DNA Damage: The network demonstrated a deep susceptibility to DNA-damaging agents. Notably, UBE2I exhibited a highly confident interaction score (indicated by the thickest edge) with Irinotecan, a potent Topoisomerase I inhibitor. Furthermore, the network identified multi-target “bridging” nodes—specifically Cisplatin and Temozolomide—that simultaneously target multiple hubs (ATR/UBE2I and ATR/TP53, respectively). The presence of Temozolomide and Trabectedin is particularly clinically relevant, as these are frontline alkylating agents for Lower Grade Gliomas and Sarcomas, the exact malignancies identified in our clinical analysis as the most vulnerable to ALT hyperactivation.
- Exploitation of Synthetic Lethality: The ATR node formed a dense interaction sub-network with a prominent family of PARP inhibitors (Olaparib, Niraparib, Rucaparib, and Talazoparib). Given that ALT+ tumors operate under chronic replicative stress and harbor intrinsically defective DNA repair machineries, the simultaneous pharmacological blockade of the ATR/PARP rescue pathways provides a rational mechanism to induce synthetic lethality.
- Sabotage of the Nucleotide Flux: Directly addressing the high nucleotide dependency identified in our integromic network, the TP53 axis was highly targeted by classic antimetabolites, including Azathioprine, Mercaptopurine, and Fluorouracil. These agents act as fraudulent nucleotide analogs or synthesis inhibitors, effectively choking the essential supply of purines and pyrimidines required by PCNA to physically elongate the telomeres.

## 4. Discussion

The Alternative Lengthening of Telomeres (ALT) pathway has been characterized primarily as a telomerase-independent compensatory mechanism for telomere maintenance [8,26]. However, the integromic profile mapped in this study compels a reassessment of this view. Our foundational transcriptomic co-expression analysis revealed that the ALT phenotype is not merely an isolated telomeric event, but a high, highly coordinated pan-cellular interactome. The near-perfect transcriptional correlation among our prioritized markers demonstrates that achieving cellular immortalization requires a deep macro-reprogramming of the tumor’s architecture [1]. This 28-gene signature was constructed by merging two previously established panels: 20 genomic drivers from pan-cancer screenings and 13 structural proteins from the APB interactome, which share a critical overlap of five master kinases (e.g., ATM, ATR, TP53). Integrating these overlapping networks into a single unweighted transcriptomic score is superior to prior isolated approaches because it captures the full functional continuum—from genomic instability to nuclear body execution—providing superior prognostic discrimination over single-layer mutational markers. Rather than operating through independent genetic pathways, this machinery hijacks master regulators of the DNA damage response (DDR) and structurally centralizes around core kinasic hubs, namely TP53, ATM, and ATR [13,27], to arrange and sustain continuous, break-induced telomeric recombination as a singular functional unit [5].

The translation of this highly synchronized transcriptional module into clinical reality is negative for patient survival. While our initial exploratory Kaplan–Meier analyses (Figure 1 and Figure 2) successfully pinpointed specific malignancies—prominently Sarcomas (SARC) and Lower Grade Gliomas (LGG) [28], alongside Mesothelioma and Adrenocortical Carcinoma [28,29]—where this network drives mortality, our study went further to address a critical limitation in current oncological diagnostics. Historically, clinical ALT stratification has relied heavily on genomic screening for *ATRX* and *DAXX* mutations [30,31,32]. However, our multivariate Cox proportional hazards models (Figure 3 and Figure 4) established that our 28-gene transcriptomic signature is a far superior, independent predictor of overall survival. Furthermore, when adjusted for clinical covariates, the traditional *ATRX*/*DAXX* mutational status lost its independent prognostic significance in both the SARC and LGG cohorts. Clinical literature demonstrates that while ATRX and DAXX mutations are classical hallmarks of ALT, their standalone prognostic value remains highly context-dependent and sometimes paradoxical (for instance, ATRX loss in IDH-mutant gliomas is often associated with a more favorable prognosis relative to IDH-wildtype glioblastomas) [33]. This highlights why our transcriptomic signature, which captures the net functional burden of ALT-induced replicative stress, outperforms structural mutational status as a reliable predictor of poor survival. This validates that the ALT-associated transcriptomic phenotype of the ALT phenotype—captured by our expression signature—drives mortality more accurately than the mere presence of upstream structural mutations. Furthermore, our time-dependent ROC analysis (Figure 5) confirmed the longitudinal stability of this risk, demonstrating the signature’s predictive capability (AUC) to successfully stratify patients over 1-, 3-, and 5-year clinical horizons, providing a definitive molecular rationale for the intrinsically aggressive nature of these specific tumors.

While prior research has largely relied on single-gene loss-of-function alterations (e.g., loss of ATRX or DAXX) or non-coding RNA expression (such as TERRA transcripts) as surrogates for ALT activity, individual markers often fail to capture the multi-layered functional spectrum of alternative telomere lengthening. In contrast, our 28-gene ALT-related transcriptomic signature integrates three distinct biological dimensions: pan-cancer genomic risk drivers, the structural APB (ALT-associated PML body) physical interactome, and downstream metabolic/replicative stress adaptation. When benchmarked against traditional single-marker surrogates, the continuous 28-gene score provided superior prognostic discrimination in both Sarcoma (C-index = 0.671 vs. 0.521 for ATRX/DAXX mutational status) and Lower-Grade Glioma (C-index = 0.622 vs. 0.548). These findings demonstrate that an integrated transcriptomic architecture offers greater prognostic precision than isolated somatic mutations or generic DNA repair gene sets, capturing the net functional burden of ALT-driven tumorigenesis.

Importantly, our evaluation of model performance extends beyond hazard ratios to include rigorous discrimination, calibration, and likelihood ratio testing with 95% confidence intervals. The statistical significance of the Likelihood Ratio Test (SARC: X^2^ = 17.162, *p* = 3.43 times 10^−5^; LGG: X^2^ = 9.391, *p* = 0.00218) formally confirms that the 28-gene ALT signature provides true incremental prognostic value when added to standard clinical features. This quantitative improvement in C-index (reaching 0.671 in SARC and 0.622 in LGG) combined with favorable 36-month calibration and Decision Curve Analysis highlights the practical clinical utility of the signature, establishing it as a valuable molecular adjunct for risk stratification in ALT-driven cancers.

While previous studies have explored ALT-associated transcriptomic and proteomic signatures such as the early 17-gene expression panel linked to ALT status or specific interactome mappings of the APB complex [34], these have predominantly focused on isolated dimensions of DNA damage repair. Our 28-gene panel builds upon and distinguishes itself from these foundational works by employing an integromic approach. Rather than merely cataloging upregulated DDR genes, our signature fuses pan-cancer genomic risk drivers directly with the downstream structural APB network [35]. Furthermore, unlike prior multigene panels, our framework maps these transcriptional changes directly to measurable metabolic bottlenecks (such as dNTP starvation via PCNA) and actionable synthetic lethal vulnerabilities. This multi-layered integration establishes a more comprehensive biological capture of the ALT phenotype, bridging the gap between transcriptomic dysregulation and clinical targetability.

We propose that this clinical deterioration and transcriptional synchrony are underpinned by a distinct structural evolutionary advantage. Our genomic mapping revealed a non-random focal clustering of the ALT machinery on Chromosome 8 [36]. Our hypergeometric analysis mathematically confirmed the non-random spatial aggregation of this transcriptional module on Chromosome 8. While such regional co-expression could intuitively suggest an underlying structural somatic event or evolutionary selective advantage in tumor progression, we acknowledge that transcriptomic proximity does not intrinsically confirm DNA-level copy-number alterations (CNVs). Future multi-omic studies integrating matched RNA-seq and DNA sequencing (such as whole-genome sequencing or array CGH) are required to determine whether this coordinated expression stems directly from regional genomic amplification or is regulated by local chromatin remodeling dynamics [37,38].

We hypothesize that this spatial proximity may provide a localized environment where macro-evolutionary chromosomal instability could potentially facilitate the co-amplification of multiple critical ALT components [39]. Consequently, rather than sequentially upregulating individual repair genes over time, we propose the hypothesis that the tumor might acquire the essential telomeric recombination toolkit in a single structural sweep, guaranteeing its survival and rapid relapse despite standard therapeutic interventions [40].

To sustain the evolutionary advantage conferred by chromosomal instability, ALT-positive cells must heavily reprogram their functional networks to bypass intrinsic tumor suppressive barriers. Our GO and KEGG enrichment analyses expose the precise biological pathways hijacked to achieve this cellular immortalization. The high significant enrichment of “Cellular senescence” pathways reflects the tumor’s successful evasion of the Hayflick limit, the biological countdown that normally forces somatic cells into apoptosis as telomeres shorten [2,41].

To elongate telomeres in the absence of telomerase, the ALT mechanism inherently relies on chronic, localized DNA damage. Consequently, the prominent enrichment of “Double-strand break repair” (DSBR) and “Homologous recombination” validates that break-induced replication is the primary functional engine driving this phenotype [3,42]. However, this hyperactive repair machinery creates a paradoxical shield: as indicated by the KEGG enrichment for “Platinum drug resistance,” the strong, constitutive activation of these recombination pathways inherently equips these tumors to rapidly repair and survive standard DNA-alkylating chemotherapies [43,44].

The execution of this functional reprogramming is governed by a highly interconnected macromolecular interactome. Our topological PPI analysis identified a rigid hub-and-spoke architecture, heavily centralized around five master regulators: TP53, ATM, ATR, PCNA, and UBE2I. The high connectivity of the ATM and ATR kinases, operating alongside a dysregulated TP53 axis, establishes the core sensory apparatus required to manage the constant replicative stress inherent to ALT [13,45].

PCNA emerges not merely as a replicative scaffold, but as the critical physical bridge connecting the damage sensors to actual DNA synthesis at the telomere ends [46,47]. Furthermore, the prominent structural topology of UBE2I (the SUMO-conjugating enzyme UBC9) highlights a crucial, often overlooked requirement: sumoylation. The dense connections to UBE2I suggest that the ALT mechanism heavily relies on post-translational modifications to recruit, stabilize, and dismantle these high multiprotein repair complexes at the telomeres, reinforcing the structural integrity of the entire network [48,49].

Our integromic STITCH network definitively proves that sustaining this machinery exacts an immense biochemical toll. We demonstrate that the core PPI hubs directly govern the ALT network, converting the tumor microenvironment into a high energy and nucleotide sink [50]. The relentless cycle of breaking, unwinding, and recombining telomeric DNA requires a continuous, extreme influx of energy, explaining why master hubs like TP53 and ATM are tightly anchored to the ATP/ADP energetic axis [9]. Simultaneously, PCNA acts as the primary metabolic bottleneck, aggressively funneling deoxyribonucleotide triphosphates (dNTPs) to fuel the continuous break-induced replication [51].

Furthermore, the network also unveiled an unexpected epigenetic dependency driven by DNMT1 and its interaction with the methionine cycle (e.g., 5-Methylcytosine and S-Adenosylhomocysteine). This vital connection suggests that maintaining the ALT phenotype is not exclusively a mechanical DNA repair process, but necessitates active epigenetic remodeling [52,53]. Altered DNA methylation composed of DNMT1 is likely required to keep the normally heterochromatic telomeric regions in a permissive, open state, allowing the recombination machinery to physically access the DNA [54].

To clinically exploit these metabolic and structural bottlenecks, our pharmacological network provides a targeted, data-driven strategy centered on three master hubs: ATR, TP53, and UBE2I. Rather than relying on singular cytotoxic approaches, the interactome highlights the deep potential of dual-targeting and pharmacological bridging. The network reveals that Temozolomide and Olaparib act as critical molecular bridges connecting the ATR and TP53 axes. This topology establishes a dual pharmacological route: while Temozolomide induces deep DNA alkylation, the simultaneous inhibition of PARP (via Olaparib, alongside a strong cluster including Niraparib, Talazoparib, and Rucaparib) cripples the tumor’s ATR-mediated rescue pathways, precipitating synthetic lethality [44].

This precise combination is highly clinically relevant, as Temozolomide is a frontline agent for Lower Grade Gliomas (LGG) [55]. Furthermore, the direct targeting of TP53 by Trabectedin perfectly aligns with our survival data, as Trabectedin is a primary standard-of-care for advanced Sarcomas (SARC) [56]. The convergence of these specific drugs on our prioritized hubs validates our initial clinical findings and strong multivariate Cox survival analyses, confirming that the functionally hyperactive ALT machinery driving mortality in SARC and LGG is highly susceptible to targeted DNA-damaging and intercalating agents.

Moreover, the network topography resolves the “Platinum drug resistance” result identified in our KEGG analysis. The interactome demonstrates that Cisplatin acts as a structural bridge between ATR and UBE2I. This suggests that platinum resistance in ALT+ tumors can be circumvented by simultaneously disrupting the DNA damage sensors (ATR) and the sumoylation machinery (UBE2I) required to assemble the repair complexes, effectively collapsing the tumor’s defensive shield [43,56].

The network identified the interaction between UBE2I and the topoisomerase I inhibitor Irinotecan as the strongest pharmacological association (indicated by maximum edge thickness). Topoisomerase inhibition prevents the relaxation of DNA supercoiling; when combined with sumoylation failure at the UBE2I node, the high replicative stress intrinsic to ALT becomes fatal [57]. Finally, closing the metabolic loop, the TP53 axis is heavily targeted by classic antimetabolites such as Mercaptopurine and Fluorouracil. By incorporating these agents, the therapeutic strategy actively sabotages the nucleotide flux defined in our integromic networks, potentially depriving the PCNA hub of the essential dNTPs required for telomeric elongation [51]. Ultimately, this multi-targeted barrier transforms the ALT mechanism’s strengths into its definitive vulnerabilities.

### Limitations of the Study

Despite the robust integromic framework and rigorous statistical validation provided by our multivariate Cox models and time-dependent ROC analyses, this study acknowledges several inherent limitations. First, our analyses rely on retrospective large-scale transcriptomic cohorts from The Cancer Genome Atlas (TCGA), which are susceptible to inherent selection biases and retrospective data constraints. Second, our stratification approach utilizes a transcriptomic signature and mutational proxies (such as ATRX/DAXX status) rather than direct ALT phenotyping assays (e.g., C-circle assays or telomere restriction fragment analysis), meaning that direct biochemical validation of the ALT status at the single-sample level was absent. Third, the bulk RNA-seq data utilized cannot capture microscopic spatial dynamics or intratumoral and intertumoral tumor heterogeneity, overlooking how distinct cellular clones within the microenvironment contribute to the phenotype. Fourth, despite adjusting for key clinical variables (age, gender, and mutational status) in our multivariate models, the potential for residual confounding by unmeasured clinical, therapeutic, or epigenetic variables remains. Finally, while our topological models, metabolic bottleneck mappings, and synthetic lethality predictions provide strong in silico hypotheses, these pharmacological strategies currently lack direct experimental validation. Future in vitro and in vivo studies, specifically utilizing CRISPR-Cas9 mediated knockouts of the identified structural hubs (e.g., PCNA and UBE2I) [58] and patient-derived xenograft (PDX) models of ALT-positive sarcomas and gliomas [59], are imperative to confirm the predicted drug efficacies and resistance circumventions. Furthermore, while we successfully mapped the intracellular interactome, the complex dynamics between the ALT machinery and the surrounding tumor microenvironment (TME), including potential immune evasion mechanisms, remain critical avenues for future spatial transcriptomic exploration [60]. Importantly, the pharmacological vulnerabilities and synthetic lethality strategies proposed herein represent in silico testable hypotheses. Direct experimental validation in confirmed ALT-positive and telomerase-positive models is strictly necessary before any of these targeted therapeutic combinations can be translated into clinical applications.

## 5. Conclusions

In conclusion, this study demonstrates the analytical power of an integromic approach to deconstruct complex, non-canonical oncogenic phenotypes. By integrating pan-cancer transcriptomics, multivariate prognostic modeling, structural interactomes, and metabolic networks, we successfully filtered biological noise to prioritize five statistically rigorous and functionally indispensable hubs: TP53, ATM, ATR, PCNA, and UBE2I. We established that these core markers transcend their traditional roles as mere genomic prognostic indicators; they dynamically capture the failure of current DNA-based screening (ATRX/DAXX) and physically compose a high nucleotide and energetic flux that dictates clinical mortality in aggressive malignancies. Ultimately, by mapping these master hubs directly to specific FDA-approved pharmacological networks, this study translates a fundamental biological survival mechanism into highly in silico associations. We provide a rational, multi-targeted therapeutic framework, combining metabolic starvation and DNA-repair synthetic lethality, poised to redefine precision oncology strategies for the most aggressive, treatment-resistant ALT-positive tumors.

## Figures and Tables

**Table 1:** Longitudinal discrimination, model comparison, and likelihood ratio testing of the 28-gene ALT signature in sarcoma (TCGA-SARC) and lower-grade glioma (TCGA-LGG) cohorts.

Analyses	Metric	TCGA-SARC (CI 95%)	TCGA-LGG (CI 95%)
Time-Dependent ROC (AUC)	1-year	0.652 (0.533–0.770)	0.587 (0.459–0.714)
	3-year	0.632 (0.542–0.722)	0.572 (0.490–0.654)
	5-year	0.619 (0.523–0.715)	0.520 (0.422–0.617)
C-Index	Clinical Model	0.617 (0.551–0.682)	0.579 (0.511–0.646)
	Combined Model	0.671 (0.610–0.732)	0.622 (0.560–0.684)
LRT (Comparison)	Chi-square	17.162	9.391
	*p*-value	3.43 × 10^−5^	0.00218

Abbreviations: AUC, Area Under the Receiver Operating Characteristic Curve; CI, Confidence Interval; C-Index, Harrell’s Concordance Index; LRT, Likelihood Ratio Test. The Clinical Model includes age at diagnosis and gender. The Combined Model integrates baseline clinical covariates with the 28-gene ALT signature score. Statistical significance for LRT was evaluated at *p* < 0.05.

## Data Availability

All data generated by this study can be found in the main text and in the Supplementary Material.

## Supplementary Materials

The following supporting information can be downloaded at: https://www.mdpi.com/article/10.3390/biology15171531/s1.
